# Developmental dynamics of stem starch accumulation in *Sorghum bicolor*

**DOI:** 10.1002/pld3.74

**Published:** 2018-08-20

**Authors:** Brian A. McKinley, Anna L. Casto, William L. Rooney, John E. Mullet

**Affiliations:** ^1^ Department of Biochemistry and Biophysics Texas A&M University College Station Texas; ^2^ Department of Soil and Crop Sciences Texas A&M University College Station Texas

**Keywords:** bioenergy, pith parenchyma, RNA‐seq, transcriptome analysis

## Abstract

Sweet sorghums were identified that accumulate up to ~9% of their total stem dry weight as starch. Starch accumulated preferentially in stem pith parenchyma in close proximity to vascular bundles. Stem starch accumulated slowly between floral initiation and anthesis and more rapidly between anthesis and 43 days post‐anthesis before declining in parallel with tiller outgrowth. Genes involved in stem starch metabolism were identified through phylogenetic approaches and RNA‐seq analysis of Della stem gene expression during the starch accumulation phase of development. Genes differentially expressed in stems were identified that are involved in starch biosynthesis (i.e., AGPase SS/LS, starch synthases, starch‐branching enzymes), degradation (i.e., glucan‐water dikinase, β‐amylase, disproportionating enzyme, alpha‐glucan phosphorylase) and amyloplast sugar transport (glucose‐6‐P translocator). Transcripts encoding AGPase SS and LS subunits with plastid localization were differentially induced during stem starch accumulation indicating that ADP‐glucose for starch biosynthesis is primarily generated in stem plastids. Cytosolic heteroglucan metabolism may play a role in stem sucrose/starch accumulation because genes encoding cytosolic forms of the disproportionating enzyme and alpha‐glucan phosphorylase were induced in parallel with stem sucrose/starch accumulation. Information on the stem starch pathway obtained in this study will be useful for engineering sorghum stems with elevated starch thereby improving forage quality and the efficiency of biomass conversion to biofuels and bio‐products.

## 1 INTRODUCTION

Plant stems transport water and nutrients to and from leaves and roots and contribute to diverse canopy architectures that influence light interception and leaf photosynthesis. Stems also serve as temporary depots for nonstructural carbohydrates such as sucrose, monosaccharides, starch, fructans, and isoprenoids (Jensen & Wilkerson, 2017; Slewinski, 2012). The C4 grasses sugarcane and sorghum are capable of accumulating large amounts and high concentrations of sucrose (Lingle, 1987; Mcbee & Miller, 1982; McKinley, Rooney, Wilkerson, & Mullet, 2016b; Murray et al., 2008). For example, the sweet sorghum Della accumulated ~31% of its stem dry weight as sucrose between floral initiation and grain maturity as well as lower levels of glucose, fructose, and starch (McKinley et al., 2016b). The large capacity for stem sucrose accumulation provides these plants with a sink for storing photosynthetically derived sucrose when requirements for metabolism, growth, and/or seed filling are satisfied. This allows continued photosynthesis with minimal feedback inhibition reducing the cost/damage associated with interception of light by leaves in the absence of a sink for energy utilization. The nonstructural carbohydrates that accumulate in stems provide a reservoir of sugars that enhance plant resilience and enable grain filling under adverse environmental conditions that cause inhibition of photosynthesis (Czedik‐Eysenberg et al., 2016). In sorghum, stem sucrose and starch levels decline in parallel with onset of tillering post‐grain maturity indicating that stem reserves also contribute to traits such as ratooning that are often associated with perennial sorghum (McKinley, Rooney, Wilkerson, & Mullet, 2016a).

Sugarcane breeders have focused on increasing the purity, concentration, and yield of sucrose in sugarcane stems (Waclawovsky, Sato, Lembke, Moore, & Souza, 2010). As a consequence of strong selection, the sucrose concentration in stems of elite sugarcane has reached an apparent plateau (~0.7 M) and recent gains in yield are correlated with increases in plant size and biomass (Waclawovsky et al., 2010; Wu & Birch, 2007). The stem sucrose concentration plateau has been attributed to constraints caused by elevated turgor associated with high sucrose concentrations in pith parenchyma cells, sucrose‐mediated feedback inhibition of photosynthesis, and carbohydrate sensing and signaling mechanisms (Wu & Birch, 2007). Sucrose concentrations in sweet sorghum are similar to sugarcane; however, as sorghum is not a large commercial source of sucrose, selection for sucrose purity has been less intense which may explain why significant levels of stem starch are observed in sweet sorghum genotypes (McKinley et al., 2016b).

Our group has been developing and designing high‐biomass sorghum hybrids that can be used for forage and production of biofuels and bio‐products (Mullet et al., 2014; Rooney, Blumenthal, Bean, & Mullet, 2007). Stems account for approximately 80% of this crop's harvested biomass; therefore, stem composition is an important attribute of biomass value and utilization. High stem sucrose yield is a useful trait for biofuels production because conversion of sucrose/sugars to biofuels and bio‐products is low cost and highly efficient (Gnansounou & Dauriat, 2010; Wu et al., 2010). However, stem sugars are unstable following plant harvest due to metabolic and microbial degradation, therefore, commercial production of sucrose from sugarcane stems requires efficient harvesting, transport, and processing (Wu et al., 2010). The instability of stem sugars prevents long‐term storage of harvested biomass restricting mill operation to times of the year when these crops can be harvested. These logistical obstacles could be reduced if sugars that are transported to stems were used to synthesize more stable polysaccharides such as starch or mixed linkage glucans. Higher stem starch could also be useful in forage crops to increase biomass digestibility. Therefore, one motivation for this study was to characterize the dynamics of stem starch accumulation during sorghum development and to increase knowledge of this pathway to enable selection and engineering.

During periods of carbohydrate surplus, leaves shunt hexoses into the synthesis of starch and starch is often the primary storage carbohydrate in tubers and grain. Starch metabolism has been extensively studied in leaves, potato tubers, rice and maize endosperms, as well as other systems (Sonnewald & Kossmann, 2013; Stitt & Zeeman, 2012). In many dicot species, starch synthesis in heterotrophic tissues involves the import G‐6‐P into plastids via the glucose‐6‐phosphate/phosphate translocator (GPT) followed by isomerization to glucose‐1‐phosphate by phosphoglucomutase (PGM). The heterotetrameric enzyme ADP‐glucose pyrophosphorylase (AGPase) catalyzes the conversion of glucose‐1‐phosphate to ADP‐glucose, the committed step in starch biosynthesis (Hädrich et al., 2011). In leaves, this reaction is rendered irreversible by the action of plastidial alkaline pyrophosphatase which hydrolyzes inorganic pyrophosphate into orthophosphate (Stitt & Zeeman, 2012). In grasses, a cytosolic AGPase has been identified that is responsible for the synthesis of 95% of the ADP‐glucose in the grain of maize (Denyer, Dunlap, Thorbjornsen, Keeling, & Smith, 1996). The cytosolic ADP‐glucose is then imported into the plastid by the action of Brittle‐1, an adenylate translocator (Shannon, Pien, Cao, & Liu, 1998). Plastid localized ADP‐glucose is then utilized by starch synthases (SS) to generate starch polymers with α‐1,4‐glycosidic linkages (Stitt & Zeeman, 2012). Following linear polysaccharide synthesis, starch‐branching enzymes (BEs) hydrolyze an oligosaccharide from the terminal end of the starch polymer just below the granule surface and attaches it deeper into the growing granule, catalyzing the formation of an α‐1,6‐linked branch point (Tetlow & Emes, 2014). This action leads to the formation of semi‐crystalline amylopectin and increases granule density, crystallinity, and stability, and the number of nonreducing ends available for chain elongation by starch synthase (Tetlow & Emes, 2014). The action of starch synthase in combination with glucan trimming by debranching enzymes (DBE)/isoamylases ISA1 and ISA2 fine tunes the structure and crystallinity of the granule and its physical properties which are compatible with species specific enzymes involved in starch degradation (Geigenberger & Fernie, 2014; Lorberth, Ritte, Willmitzer, & Kossmann, 1998; Tetlow & Emes, 2014).

The synthesis of starch creates a molecule that is highly stable (Hejazi et al., 2008). Because of this stability, industrial processing and degradation of starch require high heat to gelatinize starch. The addition of heat adds thermal energy to the polysaccharide chains of the molecule increasing the physical space between them allowing for solvation followed by hydrolysis by starch hydrolytic enzymes (Biliaderis, Maurice, & Vose, 1980). As plants cannot utilize heat to disrupt granule crystallinity, destabilization in vivo is thought to be achieved by increasing the negative charge density of the granule surface through phosphorylation thereby disrupting crystallinity and separating the polymer chains which increases enzyme accessibility (Hejazi et al., 2008). Phosphorylation of the granule surface occurs initially by glucan‐water dikinase (GWD) which phosphorylates the C‐6 position of terminal glucose monomers, followed by phosphorylation by phosphoglucan‐water dikinase (PWD) which phosphorylates the C‐3 positions of different glucosyl residues (Ritte et al., 2006). Phosphorylation by PWD is strictly dependent on prior phosphorylation by GWD (Ritte et al., 2006). After the localized swelling introduced by GWD and PWD, a phosphoglucan phosphatase encoded by *SEX4* subsequently removes the phosphate groups as these groups impede the accessibility of starch degradation enzymes to the granule surface (Oa et al., 2009). Hydrolysis begins once the reducing ends of the amylopectin chains are accessible to β‐amylases (exo‐amylases that liberate maltose). One member of the debranching enzyme family, ISA3, seems to be involved in hydrolysis of branch points facilitating degradation (Delatte et al., 2006). Additional degradation of oligosaccharides liberated from the granule is performed by the α‐amylases (endo‐amylases) and disproportionating enzymes (DPE1 and DPE2) (Critchley, Zeeman, Takaha, Smith, & Smith, 2001). Transport of glucose and maltose released by starch degradation from the amyloplast can occur through glucose transporters (pGlcT) and maltose exporters (MEX1) (Stitt & Zeeman, 2012).

The results of this study showed that sweet sorghum genotypes can accumulate up to 9% of their stem biomass as starch and that starch accumulates primarily in stem pith parenchyma. Stem starch accumulated slowly between floral initiation and anthesis then more rapidly until peak levels were reached 43 days post‐anthesis. RNA‐seq analysis of stem gene expression during this developmental time course in conjunction with homology and phylogenetic analysis of gene families involved in starch metabolism enabled the identification of genes that are likely involved in stem starch accumulation and turnover.

## 2 MATERIALS AND METHODS

### 2.1 Harvest of diverse sorghums post‐grain maturity for starch quantification

The survey of starch in sorghum [*Sorghum bicolor* (L.) Moench] stems after grain maturity was conducted from plants grown in the experimental field plots in College Station, Texas in 2014. Before planting, ammonium nitrate (32‐0‐0) was applied at a rate of 140 kg N/ha. Samples were harvested when all plants had passed grain maturity. The genotypes in this study were at variable stages of post‐grain maturity development. Three plants per plot were harvested, avoiding the end of the plots to avoid edge effects, and these three plants were used in subsequent analyses. After harvesting, leaves and leaf sheaths were removed from the stems. A stem section spanning three internodes of which the middle internode was located at 50% of the total stem length was removed by excision at the nodes. These internode sections were cut into smaller sections and dried in a forced air oven at 60°C for 3 days; the tissue was coarsely ground in a Wiley Mill (Thomas Scientific, Inc.) and subsequently finely ground in a Cyclone Sample Mill (Udy Corporation, Fort Collins, Colorado, USA) until the tissue exhibited the consistency of a powder. Then, this powered tissue was used for starch quantification.

### 2.2 Harvest of the sweet sorghum Della during development for NSC quantification

Della (Reg. no. CV‐130, PI1566819), a sweet sorghum developed from a cross of Dale and ATx622 (Harrison & Miller, 1993), was planted at the Texas A&M field station near College Station, Texas in 2012 and 2013. Before planting, ammonium nitrate (32‐0‐0) was applied at a rate of 140 kg N/ha. In both years, the experimental design consisted of two ranges of 10 plots each of Della planted in adjacent regions of the field. Additionally, two boarder rows were planted on either side of the experiment to prevent edge effects. The spacing between rows was 76 cm, and plants were thinned to 10‐cm spacing at the five‐leaf stage. Basal tillers were removed until anthesis when basal tillering ceased. Tiller removal was performed to maintain a planting density of one main culms per 10 cm. During the experiment, plants were irrigated as needed. Five plants were harvested from each experimental replication for a total of 10 plants harvested per time point for a total of 10 plants per sample. Plants were harvested a minimum of two feet from the end of the plot to avoid edge effects. After harvesting, leaves and leaf sheaths were removed from the stems. A three internode section, of which the middle internode was located at 50% of the total stem length, was removed by excision at the node. These internode sections were cut into smaller sections and dried in a forced air oven at 60°C for 3 days. Next, the internode tissue was coarsely ground in a Wiley Mill (Thomas Scientific, Inc.) and subsequently finely ground in a Cyclone Sample Mill (Udy Corporation, Fort Collins, Colorado, USA) until the tissue exhibited the consistency of a powder. Then, this powered tissue was used for NSC quantification.

### 2.3 Della stem tissue harvest for RNA‐seq

To obtain tissue for RNA‐seq, Della was grown in a greenhouse in College Station during the fall of 2011. The day length was maintained at 14 hr, and the mid‐day PAR of the green house was ~1,100–1,200 μE/m^2^/s. Plants were germinated in five gallon pots containing Metromix potting soil. Osmocote was added to the soil at the beginning of the experiment, and additional fertilization was supplemented with Peter's nutrient solution every 30 days. The plants were watered with the use of an automated watering system to eliminate the possibility of drought stress. Leaves were numbered as they appeared insuring that internode 10 was harvested at each of the eight sampling dates. Three biological replicates were harvested in the morning on each harvest date. To harvest internode samples, leaves and leaf sheaths were removed from the stem and Internode 10 was excised at the nodes to insure that a complete internode was harvested for analysis. The internode was quickly sectioned into smaller pieces with a razor for storage and ease of processing after freezing. The internode sections were placed into a 50‐ml conical tube, frozen in liquid nitrogen, and stored at −80°C until processing. To prepare tissue for RNA extraction, the frozen tissue of each internode was ground to a fine powder using a mortar and pestle and stored in 50‐ml conical tube until extraction.

### 2.4 Soluble carbohydrate quantitation

To quantify the abundance of stem nonstructural carbohydrates, previously, oven dried samples from field experiments were redried overnight using a forced air oven at 60°C to insure accuracy of dry weight measurements. Next, 200 mg of finely ground biomass was weighed (±0.5 mg) using an analytical balance and transferred to a 15‐ml conical tube. Alternatively, to quantify nonstructural carbohydrates in tissue that gave rise to the RNA‐seq data, approximately 0.5 g of frozen, pulverized tissue was placed into a previously weighed 15‐ml conical tube and lyophilized until desiccated. The tube and tissue were reweighed, and tube weight subtracted to obtain the tissue weight. Both tissue types were subjected to the following extraction protocol. Water‐soluble NSCs were extracted in 10 ml of water/Na azide (200 mg/L) solution at 50°C for 48 hr with agitation. This length of incubation was experimentally determined to be optimal for this experimental set‐up. This extraction time allowed the extraction solvent to fully penetrate all of the biomass particles and extract the soluble carbohydrates. Aliquots of 20 μl were diluted 50 ×  into 980 μl of deionized water. All HPLC samples were filtered using 0.45‐μm cellulose acetate sterile filters. Sucrose, glucose, and fructose concentrations were quantified using high performance anion exchange‐pulsed amperometric detection (HPAE‐PAD) with a Carbopac PA1 analytical column (Dionex, Sunnyvale, CA) as well as a Borate trap (Dionex) and an Aminopac column (Dionex) to reduce borate and amino acid interference (Murray et al., 2008). A solution of 75 mM NaOH was made from 50% NaOH solution (Sigma‐Aldrich, St. Louise, MO, USA) instead of pellets to eliminate carbonate contamination. This running buffer was vacuum degassed overnight and stored under a helium bed for the duration of the chromatographic run. The standard curve was validated by the incorporation of curve validation samples of known concentration throughout the experiment in accordance with the NREL LAP (Sluiter, Ruiz, Scarlata, Sluiter, & Templeton, 2008).

### 2.5 Starch quantification

The starch in stem biomass was quantified using the NREL Laboratory Analytical Procedure for extracting starch from solid biomass (Sluiter & Sluiter, 2005). After the soluble nonstructural carbohydrate extraction, samples were washed four times in methanol‐chloroform‐water extraction solvent at room temperature for 15 minutes then centrifuged at 1,640 ***g*** for 15 min to sediment structural biomass and starch granules. After the MCW washes, the samples were oven dried at 65°C for 48 hr. Next, DMSO was added to the dry samples and incubated overnight. Overnight imbibition insures that the DMSO, the gelatinization solvent, has fully penetrated the biomass and is capable of fully gelatinizing the starch which is essential to fully digest all the starch. After imbibition, the samples were incubated at 100°C for 10 min with agitation every two minutes to gelatinize the starch. Then, MOPS buffer and thermostable α‐amylase were added to digest the starch to maltose at 95°C for 1 hr with agitation to insure full digestion. Subsequently, sodium acetate buffer and amyloglucosidase were added to hydrolyze the maltose to glucose at 55°C for 24 hr. To insure that starch was fully digested, digestion completion samples were included in the assay and sampled after digestion with amylase and amyloglucosidase and stained with Lugol's iodine solution. Glucose was quantified with high performance anion exchange‐pulsed amperometric detection (HPAE‐PAD) chromatography with the use of a Carbopac PA1 analytical column (Dionex, Sunnyvale, CA). Curve validation samples were randomly interspersed among the analytical samples during chromatography to validate the glucose, fructose, and sucrose standard curves. The percentage of starch in the dry biomass was calculated from the glucose released during digestion.

### 2.6 Stereomicroscopy of internode cross sections

To visualize the location of starch in the stem by stereomicroscopy, field grown plants were harvested at anthesis and post‐grain maturity. A 1‐mm slice of stem tissue was excised with a razor blade from the middle of internode 11. These 1‐mm internode sections were infused with Lugol's solution in a vacuum. After infusion, the internode sections were stored in this solution overnight. Excess staining solution was removed by incubating the tissue slice in water for 24 hr, changing the solvent every 6 hr. The sections were imaged under white light using a Zeiss M^2^Bio Fluorescence Combination Zoom Stereo/Compound Microscope coupled with a Zeiss AxioCam digital camera (Kramer Scientific). Photographs were captured using the Zeiss AxioVision 3.0.6 software.

### 2.7 Template preparation and sequencing

RNA extraction was performed using the Trizol extraction method (https://www.mrcgene.com/rna-isolation/tri-reagent). The high salt precipitation step was included because of the potential high concentration of soluble carbohydrates in the sample. Total RNA was further purified using the RNeasy kit (Qiagen, Venlo, the Netherlands) which isolates RNAs >200 nucleotides in length. An on column DNase1 digestion was performed using the RNase‐free DNase set (Qiagen, Venlo, Netherlands) to remove any contaminating DNA. The quality of the RNA libraries was assessed using an Agilent Bioanalyzer. The mean RNA integrity number (RIN) from this quality control step was 8.9, and the minimum RIN was 8.3 indicating that the quality of the RNA libraries was high. RNA libraries were prepped for sequencing using the Illumina TruSeq^®^ V2 mRNA Sample Preparation Kit (Illumina, San Diego, California, USA), which selects for polyadenylated RNA and excludes rRNA and nonpolyadenylated RNAs from the sample. The 24 libraries were sequenced on one lane of an Illumina HiSeq 2500 in single‐end mode. The average sequence depth was 5.8 M reads.

### 2.8 Transcriptome analysis

The 70 bp reads were aligned to the *Sorghum bicolor* V3.1 genome using the HISAT2 aligner (Daehwan, Langmead, & Salzberg, 2015; McCormick et al., 2018). Expression was quantified using the StringTie version 1.3 software (Pertea et al., 2015). Analysis of gene expression was performed on TPM normalized data. The prepDE.py script was used to convert nucleotide coverage data from StringTie into reads that could be used by differential expression statistical packages that use conventional raw reads. Differential expression and the FDR‐adjusted *p*‐values were calculated using the edgeR package. The fold‐change displayed in the tables is the absolute value of the maximum fold‐change value between any two time points. The FDR‐adjusted *p*‐value represents the *p*‐value associated with the maximum fold‐change values shown in the gene tables. In some cases, zero expression at one time point, when being compared to another time point, led to a very high differential expression value. To minimize this effect in instances with very high DE values, the lowest time point was considered to be a TPM = 1 and, therefore, the fold‐change was recalculated as FC = maxTPM/1 (McKinley et al., 2016b). These instances are indicated with the “>“sign in front of the fold‐change values in the gene tables as the actual fold‐change is assumed to be some value greater than the value displayed. Functional analysis of gene families utilized gene functional annotations generated by DOE‐JGI for the *Sorghum bicolor* V3.1 genome available through Phytozome (https://phytozome.jgi.doe.gov/pz/portal.html#!info?alias=Org_Sbicolor) (McCormick et al., 2018).

### 2.9 Phylogenetic analysis of gene families to identify orthologs

Phylogenetic analysis was used increase the accuracy of gene family annotation. This approach was based on the identification of genes in other species with known previously validated function. The primary protein sequences were obtained Phytozome or NCBI. The protein sequences of these validated genes and the sorghum candidate homologs were then clustered using the CLUSTAL Omega algorithm using default settings.

### 2.10 Identification of chloroplast targeting peptide sequences

Peptide sequences were obtained from Biomart, hosted by the Phytozome database. The primary transcript was used for the analysis. The ChloroP algorithm (http://www.cbs.dtu.dk/services/ChloroP/) was used to predict the presence of chloroplast targeting sequences in peptides sequences.

## 3 RESULTS

### 3.1 Variation in sorghum stem starch accumulation in diverse sorghum accessions

**Figure 1:**
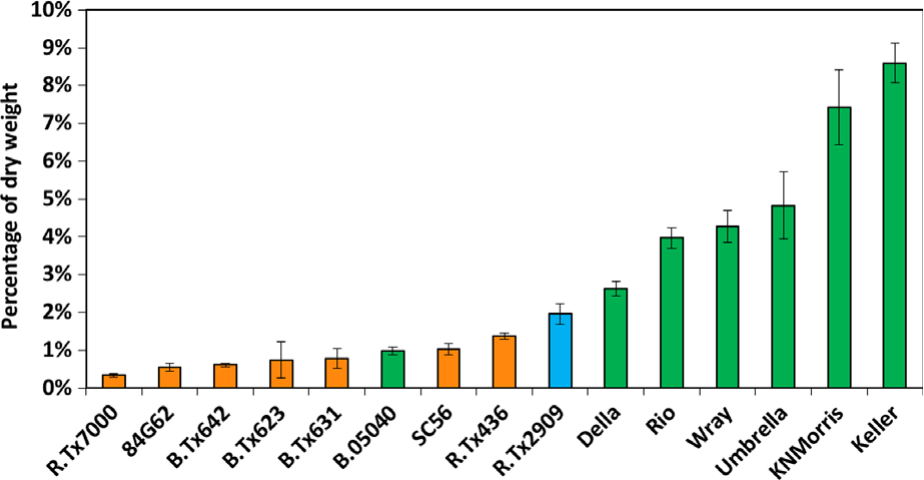
A survey of the percent composition of starch in the stem of diverse sorghum genotypes grown in the field in College Station, TX and assayed after grain maturity. Genotypes represented by green bars are sweet sorghums, orange bars represent grain sorghums, and the blue bar represents a forage sorghum. Error bars represent *SEM*

The level of starch present in stems of field grown grain and sweet sorghum genotypes was analyzed after grain maturity. A stem segment spanning three internodes located approximately mid‐stem was assayed for levels of starch and other nonstructural carbohydrates. The sorghum genotypes analyzed accumulated 0.2% to ~9% of their internode biomass as starch (Figure 1). Stem starch levels were generally higher in sweet sorghum genotypes (Figure 1, green bars) compared to grain sorghum genotypes (Figure 1, orange bars).

### 3.2 Developmental dynamics of starch accumulation in Della sorghum stems

**Figure 2:**
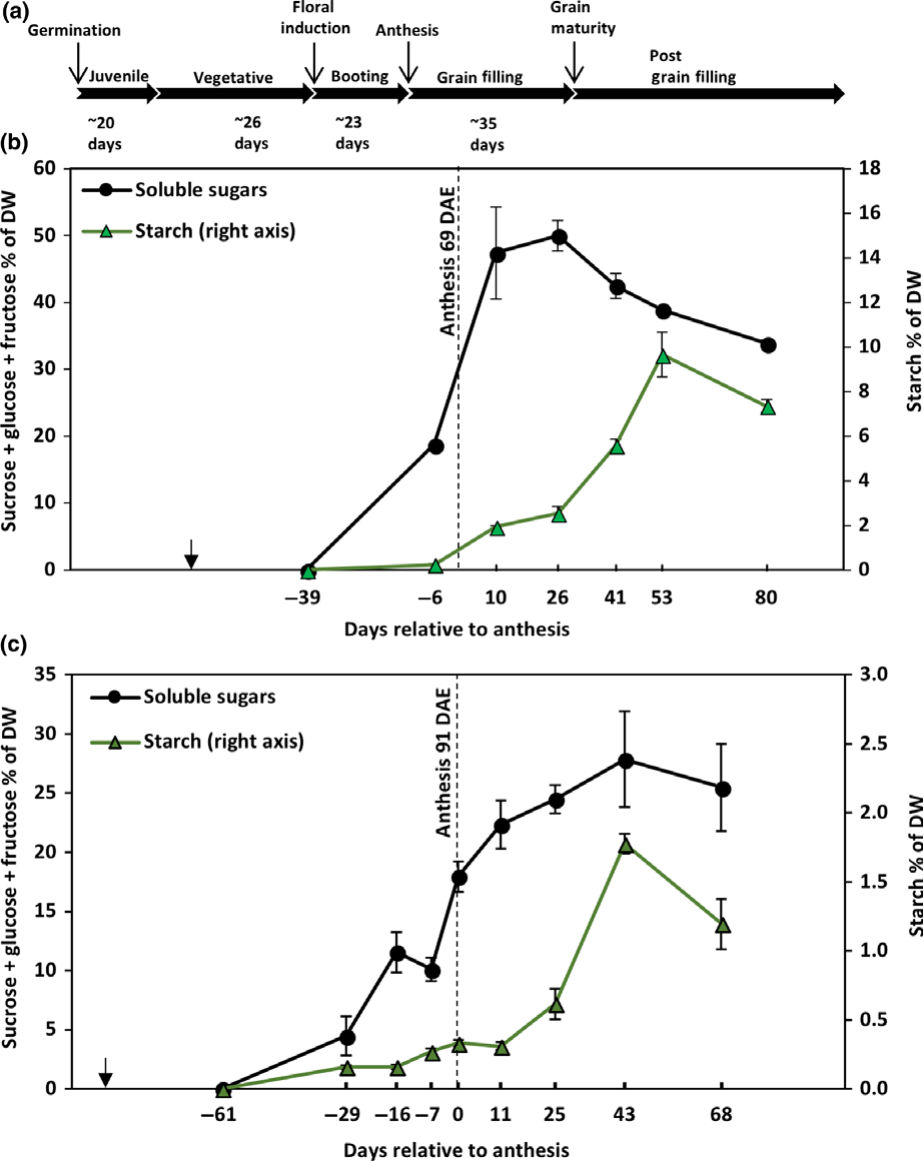
Temporal pattern of starch accumulation in the stem of the sweet sorghum Della in field and green house environments. (a) Timeline of Della development in the field in College Station, TX, in 2011. (b) The accumulation profile of total soluble sugars (sucrose + glucose + fructose) and starch in the Della stem in the field in College Station, TX, 2011. The dotted line represents anthesis. The *x*‐axis represents days relative to anthesis. Negative numbers are days prior to anthesis, and positive numbers are days after anthesis. The black arrow represents seedling emergence, that is, the start of the experiment. (c) The accumulation profile of total soluble sugars (sucrose + glucose + fructose) and starch in the Della stem in the greenhouse in College Station, TX, Fall, 2011. Error bars represent*SEM*

Sorghum progresses through a juvenile and vegetative phase following germination (Figure 2a). The reproductive phase begins with floral initiation, followed by anthesis approximately 25–30 days later, grain maturity and a post‐grain maturity phase where tillering often occurs (Figure 2a). The sweet sorghum Della grown in the field in College Station reached anthesis 69 days after emergence (DAE), and grain maturity was reached approximately one month after anthesis. The developmental time course of nonstructural carbohydrate accumulation in the stems of the sweet sorghum Della was characterized in field grown plants during the summers of 2012 and 2013 (Figure 2b). The analysis revealed that Della stems accumulated starch post‐anthesis and that peak stem starch levels were reached after grain maturity. Stem starch accumulated at its highest rate from 26 days post‐anthesis to ~53 days post‐anthesis (0.26% of internode dry biomass/day). Starch accounted for ~9.5% of Della's stem dry weight 53 days after anthesis. In contrast, stem soluble sugar levels reached a maximum ~26 days post‐anthesis and remained high until 53 days post‐anthesis (Figure 2b). Between 53 and 80 days after anthesis, levels of stem sucrose and starch decreased in parallel with leaf senescence and apical tiller outgrowth. Similar results were obtained when Della was grown in the greenhouse (Figure 2c). When Della was grown in the greenhouse to facilitate RNA‐seq analysis, the developmental time course of soluble sugar and starch accumulation in stems was similar to plants grown in the field (Figure 2c). However, in greenhouse grown plants, soluble sugar and starch levels in Della stems were lower than in field grown plants likely due to lower light levels in the greenhouse.

### 3.3 Distribution of starch in stems

**Figure 3:**
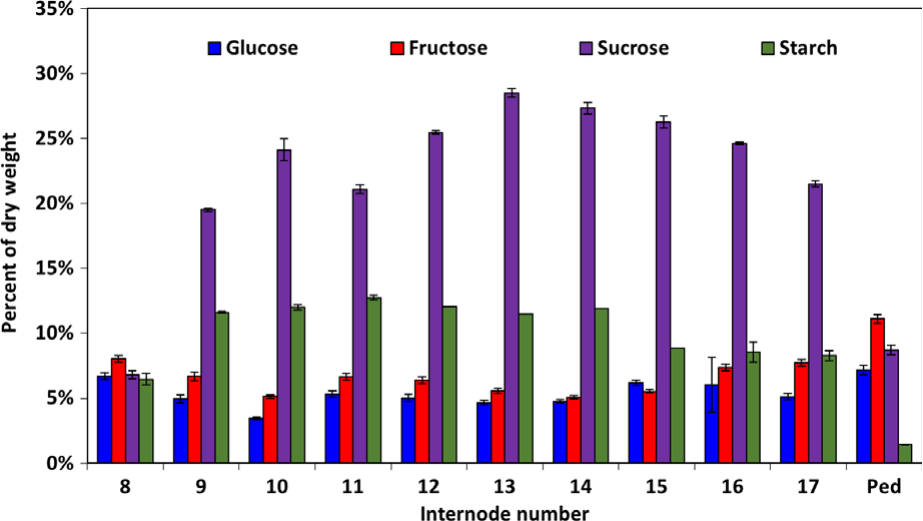
The distribution of nonstructural carbohydrates in the sweet sorghum stem as a percentage of the total dry biomass of each internode. Internode number increases proceeding up the stem. Ped, peduncle. Error bars represent*SEM*

The amount of starch and soluble carbohydrates in each of the elongated internodes of Della stems was assayed at grain maturity (Figure 3). Most of the stem internodes had similar nonstructural carbohydrate concentration profiles. However, sucrose concentrations were lower (and monosaccharides somewhat higher) in internodes located near the base of the plant (internode 8), internodes just below the peduncle (internodes 16, 17), and the peduncle. Excluding the peduncle, starch accounted for >5% of the internode dry biomass of every internode at grain maturity and internodes 9–11 has the highest starch levels (~12.5% of dry weight) (Figure 3).

**Figure 4:**
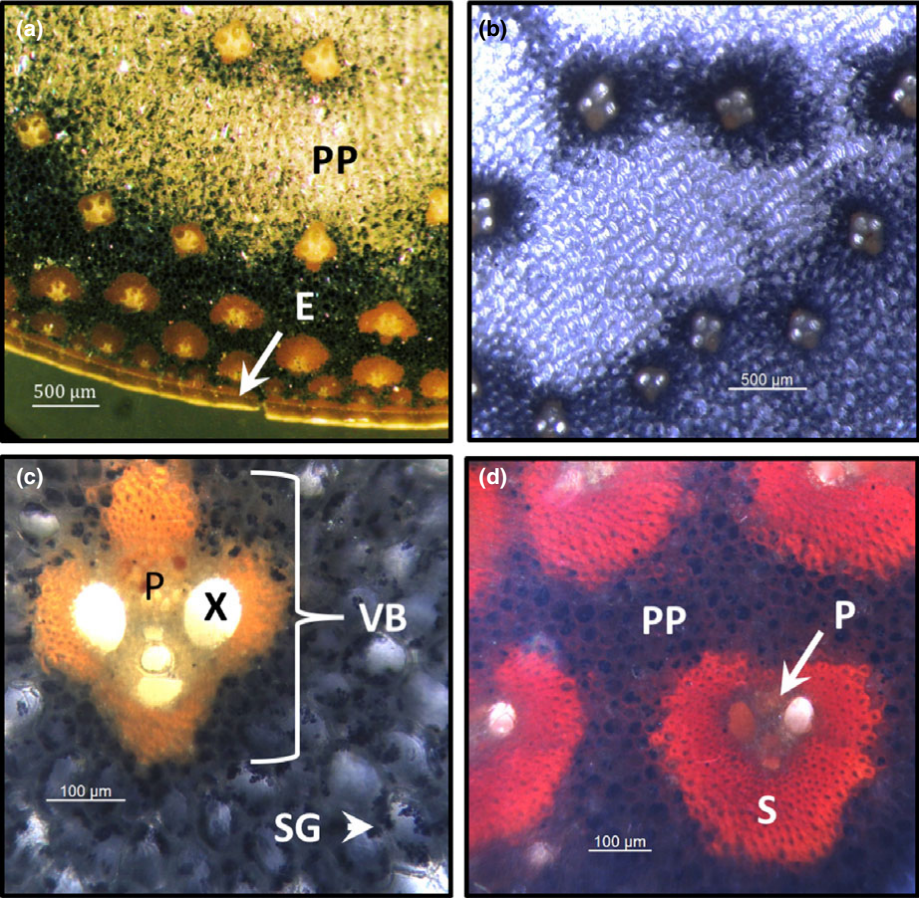
Localization of starch within sweet sorghum Della internodes visualized using stereomicroscopy. Starch was stained with Lugol's solution through vacuum infiltration. (a) Low resolution image of an internode cross section that shows the distribution of starch in the internode. (b) Image of vascular bundles of the central region of the Della internode. (c) High‐resolution image of a vascular bundle located near the central region of the internode. (d) High‐resolution image of a vascular bundle located in the rind region at the periphery of the internode near the epidermis. PP, pith parenchyma; SG, starch granule; E, epidermis; S, sclerenchyma sheath; P, phloem; X, xylem

The radial distribution of starch across stem internodes was assayed by infusing cross sections with Lugol's iodine solution which stains starch dark blue prior to visualization using a stereo‐microscope (Figure 4). This analysis revealed that starch accumulated at relatively high levels in pith parenchyma cells in regions of high vascular bundle density (Figure 4a). Starch levels were also elevated in pith parenchyma cells that surround vascular bundles in the central portion of the stem and decreased with increasing distance from the vascular bundles (Figure 4a and b). Starch granules were not observed within cells of the vascular bundles; however, this tissue was tinted blue after staining likely because of the scattering of blue light through these cell types from surrounding starch‐containing cells (Figure 4c and d). High levels of starch were not observed in the epidermis of the stem, but starch accumulated in cells just beneath the epidermis (Figure 4a).

### 3.4 Expression of genes involved in stem starch metabolism during plant development

One overall goal of this study was to identify and characterize the expression of genes involved in stem starch metabolism. The increase in starch accumulation in stems between floral initiation and 53 days post‐anthesis provided an opportunity to identify genes in starch metabolism that show increased expression in parallel with stem starch accumulation. Data on stem gene expression were obtained by extracting RNA for RNA‐seq analysis from stem internodes of Della plants grown in the greenhouse at eight times between floral initiation (~46 DAE) through 80 days post‐anthesis (~175 DAE). Genes previously identified in other plants that are involved in starch synthesis, turnover, and plastid sugar transport were used to identify sorghum homologs based on sequence similarity and phylogenetic analysis of gene families (Supporting information Tables S1–S3). Sorghum encodes a family of genes for most steps in starch metabolism, and specific genes in each family were differentially expressed in stems in parallel with starch accumulation.

**Figure 5:**
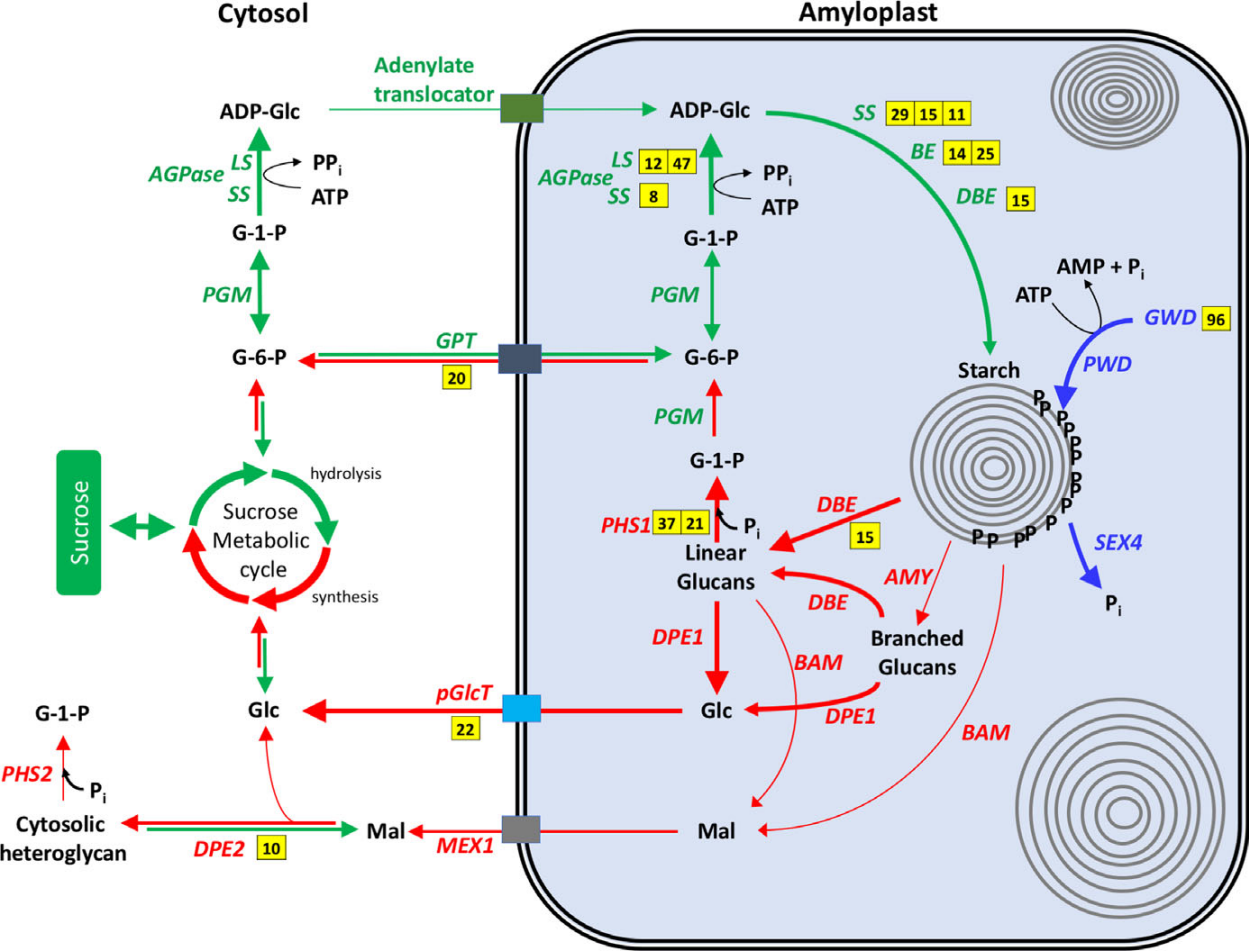
Differentially expressed genes of starch metabolism in the context of their pathways. Yellow boxes represent the fold‐change of differentially expressed genes of the pathway. Selection criteria: 10‐fold induction after anthesis with maximum expression >20 TPM. Thick lines represent hypothetical pathway flux. AGPase; ADP‐glucose pyrophosphorylase, LS; large subunit (of AGPase), SS; small subunit (of AGPase), PGM; phosphoglucomutase, GPT; glucose‐6‐phosphate/phosphate translocator, SS; starch synthase, BE; branching enzyme, DBE; debranching enzyme, GWD; glucan‐water dikinase, PWD; phosphoglucan‐water dikinase, SEX4; phosphoglucan phosphatases, AMY; α‐amylase, BAM; β‐amylase, DPE; disproportionating enzyme, PHS; α‐glucan phosphorylase, MEX1; maltose transporter, pGlcT; glucose transporter

To help visualize patterns of gene expression in the context of the entire starch pathway, a working model of stem starch metabolism was developed based on prior knowledge of this pathway obtained in other plants/organs (Smith, Denyer, & Martin, 1997; Stitt & Zeeman, 2012). In the model, genes encoding enzymes that are likely involved in stem starch synthesis are highlighted in green, genes encoding proteins involved in starch turnover are highlighted in blue or red, and those encoding proteins involved in sugar transport through the outer plastid membrane are shown in colored boxes (Figure 5). Genes expressed in stems at >20 TPM and that were induced >fivefold in parallel with stem starch accumulation between floral initiation and grain maturity have information on fold‐induction presented in yellow‐highlighted boxes adjacent to each gene. This overview shows that genes encoding proteins involved in starch biosynthesis (i.e., plastid *AGPase, SS, BE, DBE*), turnover (i.e., *GWD*,* DBE*,* DPE*,* PHS*), and transport (i.e., *GPT*,* pGlcT*) were upregulated between floral initiation and 43 days post‐anthesis in parallel with stem starch accumulation.

### 3.5 Expression of genes encoding AGPases

**Figure 6:**
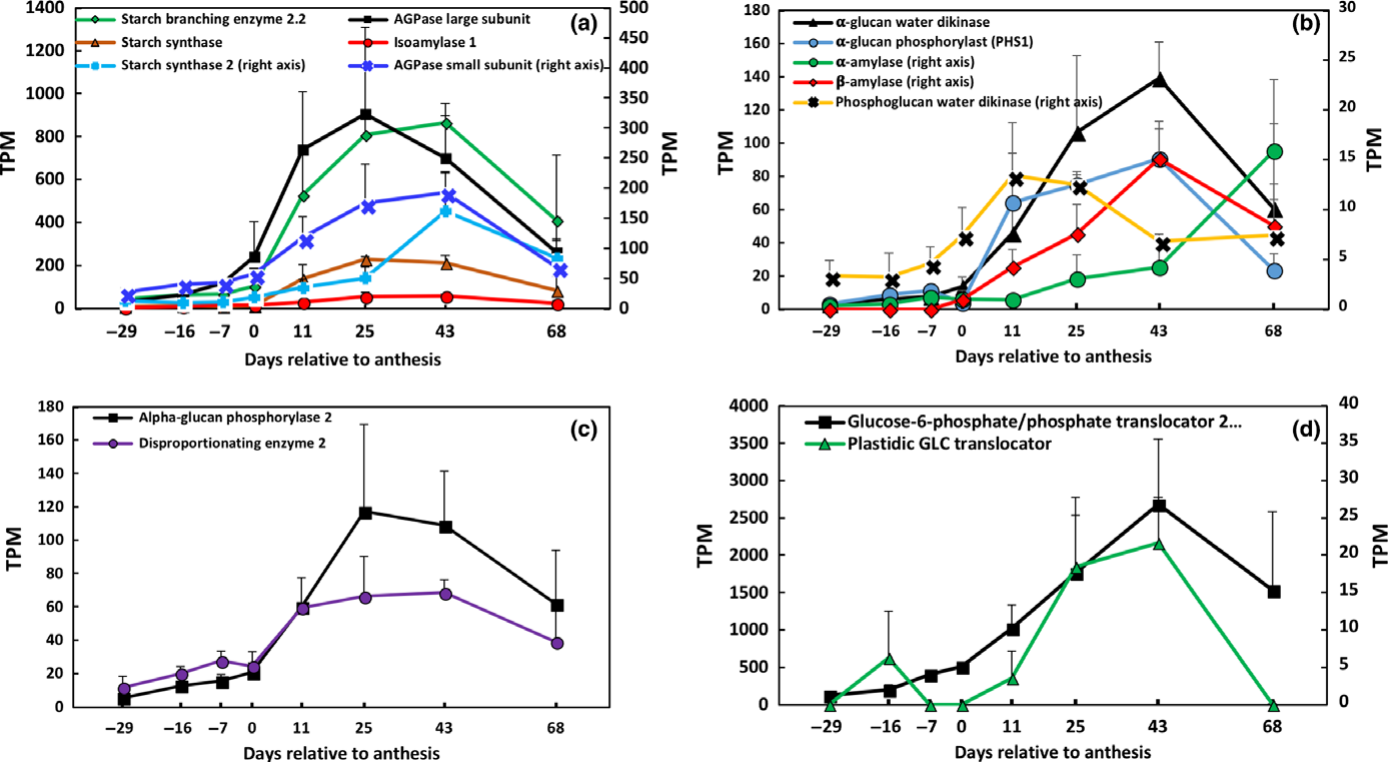
Expression profiles of starch metabolism genes in the stem that are induced after anthesis. (a) Starch biosynthesis genes. (b) Starch degradation genes. (c) Expression of starch metabolism genes localized outside of the amyloplast. (d) Transporters potentially involved in import and export of sugar into the amyloplast. Error bars represent *SEM*

Two sorghum genes encoding AGPase small subunits (SSs) and four genes encoding AGPase large subunits (LSs) were identified in the sorghum genome (Supporting information Figure S1; Supporting information Table S1). Several different transcripts were produced from these genes. One gene encoding AGPase SS (Sobic.002G160400, *APS1*) and one gene encoding AGPase LS (Sobic.009G245000; *APL2*) were expressed at lower levels in stems at floral initiation and at gradually increasing levels during development with peak expression between 25 and 43 days post‐anthesis (Figure 6a; Supporting information Table S1). Sobic.009G245000 (*APL2*) was differentially expressed 47‐fold higher in stems post‐anthesis and Sobic.002G160400 (*APS1*) eightfold higher (Figure 6a; Supporting information Table S1). Both of these genes were expressed at ~five‐tenfold lower levels in leaves compared to stems; however, expression in panicles was similar to stems (Supporting information Figure S1).

Many grasses synthesize some portion of the ADP‐glucose used for starch synthesis in grain in the cytosol (Beckles, Smith, & Ap Rees, 2001). As the stem post‐anthesis is a heterotrophic carbohydrate storage organ similar to grain (Emanuelsson, Nielsen, & von Heijne, 1999), an analysis was conducted to characterize the expression of genes/transcripts involved cytosolic ADP‐glucose synthesis during development (Supporting information Figures S1 and S2). Alignment and phylogenetic analysis of sorghum and maize AGPase gene homologs that are localized in the cytoplasm or plastid (Huang, Hennen‐Bierwagen, & Myers, 2014) and ChoroP prediction of plastid transit sequences (Xuan et al., 2013) were used to identify sorghum transcripts that encode AGPase subunits that would localize in the cytoplasm. This analysis identified two transcripts (Sobic.007G101500.2, Sobic.007G101500.3) that encode AGPase small subunits and three transcripts (Sobic.003G230500.1/.2/.3) that encode AGPase large subunits predicted to produce proteins that localize in the cytoplasm (Supporting information Figure S1). These transcripts are expressed at lower levels in stems compared to other genes/transcripts that encode AGPase subunits with predicted plastid localization. Taken together, this indicates that a plastid localized AGPase is the primary source of ADP‐glucose for starch synthesis in stem amyloplasts.

### 3.6 Genes encoding stem starch synthases and starch‐branching/debranching enzymes

ADP‐glucose is the substrate of starch synthases, the enzyme family responsible for adding glucose monomers to the reducing ends of growing amylose and amylopectin chains (Hirose & Terao, 2004). The starch synthase gene family consists of 10 genes in rice and maize (Supporting information Table S1). This family can be further divided into the soluble and granule bound starch synthases, with each of these groups containing unique subtypes (Supporting information Figure S3; Supporting information Table S1) (Hirose & Terao, 2004). Due to the functional complexity of the family, a phylogenetic analysis was conducted to align sorghum gene annotations with gene family members identified in rice and maize (Supporting information Figure S3) (Hirose & Terao, 2004). This analysis identified ten genes in the sorghum, the same number of genes in the rice starch synthase gene family (Supporting information Figure S3) (Hirose & Terao, 2004). Eight of the 10 genes in the family were expressed in sorghum stems. Most of these family members showed peak expression in stems post‐anthesis (Supporting information Table S1). A gene encoding *GRANULE BOUND STARCH SYNTHASE* (*GBSS*), Sobic.002G116000.1, was the most highly expressed gene in the family (max TPM = 737), and the gene was differentially expressed in stems between floral initiation and 43 days post‐anthesis (DE = 11‐fold) (Supporting information Table S1). The second most abundantly expressed gene of this gene family was a *STARCH SYNTHASE I* (max TPM = 229, DE = 29‐fold), and the third was a *STARCH SYNTHASE II* (max TPM = 162, DE = 15‐fold) (Figure 6a).

The starch‐branching enzymes and debranching enzymes tune starch granule structure to achieve specific physicochemical properties of the granule (Smith et al., 1997). Four genes encoding starch‐branching enzymes were expressed in the sorghum stem with peak expression after anthesis (Supporting information Table S1). The most highly expressed gene/transcript was Sobic.006G066800.2 (max TPM = 865, 43 days after anthesis) a gene that was also differentially expressed during plant development (DE = 25‐fold) (Figure 6a). Isoamylase 1‐3 are starch‐debranching enzymes. Isoamylases 1 and 2 are involved in starch biosynthesis (Supporting information Table S1), tuning the structure of amylopectin to facilitate crystallization (Hussain et al., 2003). Evidence suggests that isoamylase 3 is involved in starch degradation (Supporting information Table S2) (Delatte et al., 2006). Three transcripts of *ISA1* and *ISA2* were expressed in stems with peak expression occurring 25 days after anthesis (Figure 6a; Supporting information Table S1). The gene encoding isoamylase 3 (Sobic.002G233600) produced three transcripts that varied in abundance in stems with peak expression occurring post‐anthesis (Supporting information Table S2).

### 3.7 Genes involved in stem starch degradation

The expression of several genes that encode enzymes involved in starch turnover increased between floral initiation and grain maturity (Supporting information Table S2). To enhance starch degradation, the crystallinity of the starch granule is reduced through the action of GWD (Figure 5). The expression of *GWD* (Sobic.010G143500.1) was induced ~96‐fold between floral initiation and grain maturity in parallel with the accumulation of starch in the sorghum stem (Figure 6b; Supporting information Table S2). There were three splice variants derived from a gene expressed in the stem that encodes PWD (Supporting information Table S2). All the *PWD* splice variants were expressed at ~10‐fold lower levels compared to *GWD,* and only one transcript was differentially expressed (~fivefold) with peak expression post‐anthesis (Table S2). A gene encoding phosphoglucan phosphorylase (*SEX4*, Sobic.001G53880) showed twofold higher expression in stems post‐anthesis (Supporting information Table S2).

Amylases and isoamylase help degrade starch grains to maltose and branched and linear glucans (Figure 5). Three genes encoding α‐amylase and ten genes encoding β‐amylase were expressed in sorghum stems. The α‐amylase gene with the highest expression was expressed at similar levels from floral initiation through grain maturity. Two other α‐amylase genes were expressed at low levels until 68 days post‐anthesis when starch levels were decreasing in stems (Supporting information Table S2). Two of the genes encoding β‐amylase were expressed at high levels in stems at floral initiation. These genes may be associated with chloroplasts located in the outer cell layers of the stem. Four members of the gene family were expressed at approximately the same level in stems during the developmental stages analyzed. In addition, four other members of the β‐*AMYLASE* gene family were expressed at relatively low levels prior to anthesis with their maximum expression peaking post‐anthesis (Figure 6b; Supporting information Table S2).

Two genes encoding DPE1 and DPE2 were significantly upregulated in stems between floral initiation and grain maturity (Figure 6c; Supporting information Table S2). *DPE2*, which encodes an enzyme with predicted cytosolic localization, was more highly expressed and upregulated ~10‐fold between floral initiation and 43 days post‐anthesis. *DPE1*, which encodes an enzyme with predicted plastid localization, was expressed at lower levels, but also induced during this phase of plant development. Two genes encoding heteroglucan phosphorylase (*PHS1, PHS2*) were expressed at similar levels, and gene expression was induced ~21‐fold and ~37‐fold, respectively, with peak expression 43 days post‐anthesis (Figure 6b and c; Supporting information Table S2). *SbPHS1* has a predicted localization in plastids whereas *SbPHS2* has a predicted localization in the cytosol (Figure 6b and c; Supporting information Table S2) consistent with analysis of these proteins in other plants (Beck & Ziegler, 1989; Fettke, Malinova, Eckermann, & Steup, 2009).

### 3.8 Differential expression of starch pathway genes in stems and leaves

**Figure 7:**
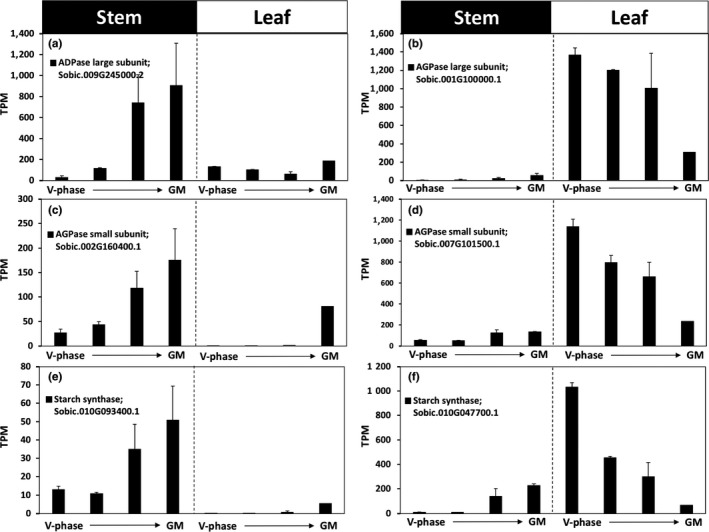
Tissue‐specific gene expression of starch biosynthetic genes. (a and b) AGPase large subunit expressed in stems and leaves, respectively. (c and d) AGPase small subunit in stems and leaves, respectively. (e and f) Tissue‐specific starch synthases expressed in stems and leaves, respectively. Error bars represent *SEM*

The expression of genes involved in starch metabolism in leaves and stems was compared to see if members of gene families were regulated in an organ‐specific manner. The expression of pairs of genes from the same gene family was compared using RNA isolated from leaves and stems at the same time in the morning during four stages of plant development (Figure 7 and Supporting information Figure S4). The analysis showed that expression of Sobic.009G245000.2 (*AGL2*) was higher in stems vs. leaves during the phase of development analyzed whereas the related family member Sobic.001G100000.1 (*AGL2*) was expressed at higher levels in leaves compared to stems (Figure 7a and b; Supporting information Figure S4). Similarly, Sobic.002G160400.1 (*AGS1*) was expressed at higher levels in stems vs. leaves whereas the family member Sobic.007G101500.1 (*AGS1*) was expressed at higher levels in leaves compared to stems during these same developmental stages (Figure 7c and d; Supporting information Figure S4). A pair of genes encoding starch synthases (Sobic.010G093400.1, Sobic.010G047700.1) showed similar divergent patterns of differential expression in leaves and stems (Figure 7e and f; Supporting information Figure S4).

### 3.9 Genes involved in stem amyloplast sugar transport

The increase in stem starch accumulation between floral initiation and grain maturity and remobilization post‐grain maturity could require higher expression of genes encoding the plastid localized glucose‐6‐phosphate/phosphate translocator (*GPT*), the maltose transporter (*MEX1*), the plastidial glucose transporter (*pGlcT*), and/or the adenylate translocator. RNA‐seq analysis showed that a gene encoding a member of the *GLUCOSE‐6‐PHOSPHATE/PHOSPHATE TRANSLOCATOR 2 (GPT2)* family was induced ~20‐fold in parallel with increased stem starch accumulation during development (Figure 6d; Supporting information Table S3). This gene was highly expressed (max TPM = 2,689) and showed strong differential expression during development (FDR‐adjusted *p*‐value = 0.00021) (Figure 6d; Supporting information Table S3). Induction of this highly expressed gene suggests that glucose‐6‐phosphate is a predominant metabolite transported to and from stem amyloplasts. A gene *PLASTIDIAL GLUCOSE TRANSPORTER* (*pGlcT*) encodes a protein that transports glucose released from starch degradation out of the plastid. This gene showed increased expression in stems post‐anthesis; however, the relative expression of *pGlcT* (max TPM = 150) was much lower than *GPT2* (TPM = 2,689) (Figure 6c, Supporting information Table S3). The plastid ATP/ADP‐transporter translocates adenylates across plastid membranes thus maintaining plastidial ATP supply (Figure 6c; Supporting information Table S3) (Tjaden, Mohlmann, Kampfenkel, Henrichs, & Neuhaus, 1998). One member of this gene family was expressed in stems (max TPM = 120) although expression did not increase in parallel with starch accumulation during development (Supporting information Table S3). The gene *MALTOSE EXCESS 1* (*MEX1*) encodes a protein that transports maltose out of plastids. This gene was expressed at low levels in stems (max TPM = 8) and was not differentially expressed between floral initiation and 43 days post‐anthesis (Supporting information Table S3).

## 4 DISCUSSION

The overall goal of this study was to learn more about the regulation of starch accumulation in sorghum stems because increasing stem starch levels could improve biomass yield and the composition of high‐biomass sorghum that is used for forage and production of biofuels and bio‐products. While prior studies have noted the presence of starch in sorghum stems (Gutjahr et al., 2013; McBee, Waskom, & Creelman, 1983; McKinley et al., 2016b), the current study showed that a number of sweet sorghum genotypes can accumulate up to ~9% of their stem dry weight as starch. The capacity for significant stem starch accumulation in sweet sorghum genotypes that also accumulate high levels of stem sucrose indicates that even higher levels starch could be generated by altering the partitioning between sucrose and starch. Moreover, nearly all of the internodes that comprise Della stems accumulated high levels of sucrose and ~10%‐12% starch. Therefore, high‐biomass sweet sorghums that accumulate 80% of their biomass in stems that grow 4–5 m in length and 20–40‐mm diameter have a large potential capacity for storing stem starch. The stems of the sweet sorghum Della have a nonstructural carbohydrate to lignocellulose ratio of ~2.8 at harvest (McKinley et al., 2016b). Increasing stem starch further could potentially improve yield by reducing feedback inhibition of photosynthesis that occurs when stems reach maximum sucrose holding capacity (Gutjahr et al., 2013; Paul & Foyer, 2001) and improve the efficiency and lower the cost of biomass conversion to biofuels and bio‐products.

Starch accumulation was highest in stem pith parenchyma cells adjacent to vascular bundles in the subepidermal region of the stem where vascular bundle density was highest. The observation that starch accumulates in this region of the stem suggests that sucrose/sugar availability is high near vascular bundles. Starch also accumulated in pith parenchyma cells immediately adjacent to vascular bundles located in the central region of the stem. Interestingly, in the center of the stem where vascular bundle density is lower, pith parenchyma cells located furthest from vascular bundles accumulated lower levels of starch suggesting that sucrose/sugars derived from the phloem are used preferentially by pith parenchyma cells closest to the phloem and that starch accumulation in stem pith cells is responsive to sucrose availability.

Leaves are the primary source of sucrose that is used for synthesis of starch in stem pith parenchyma cells. Prior studies have examined the transport of sucrose from sorghum leaf mesophyll cells to stems (Sowder, Tarpley, Vietor, & Miller, 1997) and identified transporters that are involved in this pathway (Bihmidine, Baker, Hoffner, & Braun, 2015; Bihmidine, Julius, Dweikat, & Braun, 2016; Milne et al., 2017; Mizuno, Kasuga, & Kawahigashi, 2016). In fully elongated internodes that are accumulating sucrose post‐floral initiation, sucrose can move from the phloem to stem storage parenchyma cells via an apoplastic and/or symplastic pathway with subsequent sequestration in pith parenchyma cell vacuoles (Bihmidine et al., 2015; Milne, Offler, Patrick, & Grof, 2015; Sowder et al., 1997; Tarpley & Vietor, 2007). Sucrose taken up by storage pith parenchyma post‐anthesis is then sequestered in vacuoles or hydrolyzed by cytoplasmic invertases or sucrose synthases generating hexoses that can be used for starch biosynthesis. The ~20‐fold induction of *GPT2* expression in stems in parallel with increased starch accumulation during development indicates that transport of G‐6‐P to and from the plastid plays an important role in stem starch metabolism.

### 4.1 Genes involved in sorghum stem starch metabolism

Sorghum genes potentially involved in stem starch metabolism were identified based on their sequence similarity to genes previously identified in other species, phylogenetic analysis of gene families, and increased expression during starch accumulation in sorghum stems between floral initiation and 43 days post‐anthesis. This approach identified sorghum genes predicted to encode enzymes involved in starch metabolism that were significantly upregulated in parallel with the accumulation of stem starch during development (Figure 5). Several genes encoding steps in starch biosynthesis were differentially expressed relative to other gene family members in stems vs. leaves during monocot development. This suggests that stem starch accumulation is regulated in part through differential expression of specific gene family members involved in starch metabolism. The basis of differential regulation and confirmation that these genes are directly involved in stem starch metabolism will require additional studies involving targeted gene modification.

In monocot grain endosperm tissue, most of the ADP‐glucose used for starch biosynthesis is synthesized in the cytoplasm and imported into amyloplasts (Beckles et al., 2001; Johnson et al., 2003). In sorghum stems that were accumulating starch, the level of transcripts encoding cytosolic versions of AGPase subunits was low and abundance did not increase in parallel with stem starch accumulation. In contrast, transcripts encoding AGPase subunits with predicted plastid localization were present at higher levels and RNA abundance increased ~eightfold (Sobic.002G1600400.1; *APS1*) and ~30‐fold (Sobic.009G245000.2; *APL2*) from floral initiation to 43 days post‐anthesis. This indicates that generation of ADP‐glucose for starch biosynthesis occurs in plastids similar to other monocot vegetative tissues. This conclusion is consistent with elevated expression of *GPT2* that could transport G‐6‐P into plastids thereby providing a source of G‐1‐P for ADP‐glucose biosynthesis. Eight genes encoding starch synthases were expressed in stems, and three of these genes showed increased expression (11–29‐fold) in parallel with stem starch accumulation between floral initiation and 43 days post‐anthesis. Two of the three genes encoding starch‐branching enzymes and a gene encoding isoamylase 1 showed similar dynamics of gene expression. Taken together, this study identified genes in gene families that encode enzymes for known steps in starch biosynthesis that are differentially expressed in parallel with stem starch accumulation.

As stem starch is a carbohydrate reserve, it was not surprising that genes encoding enzymes involved in starch turnover were induced in parallel with stem starch accumulation. A gene encoding alpha‐glucan‐water dikinase that mediates an early step in starch turnover showed the highest differential expression during the developmental time course (~96‐fold). Expression of starch phosphorylase (*PHS1*), which generates G‐1‐P from linear malto‐oligosaccharides released from the starch granule, was induced 37‐fold. Subsequent conversion of G‐1‐P to G‐6‐P would facilitate export of hexoses from the plastid via GPT2. Genes encoding other enzymes involved in starch turnover were induced to lower extents. The expression patterns of the ten gene family members encoding β‐amylase that were expressed in stems was especially complex, with some family members differentially expressed in stems at floral initiation followed by decreased expression, and others showing differential expression post‐anthesis. Further studies on the biological function of this complexity are warranted.

Genes encoding the disproportionating enzyme (DPE) and glucan phosphorylase (PHS) were expressed in stems. These genes encoded proteins with predicted plastid localization (DPE1, PHS1) as well as proteins with predicted cytosolic localization (DPE2, PHS2). Prior studies have shown that the cytosolic forms of these enzymes are involved in the synthesis/turnover of cytosolic heteroglucans (Fettke et al., 2005, 2009). The function of the cytosolic heteroglucans has been investigated using knock‐outs/RNAi constructs targeting *PHS2* and *DPE2* (Chia et al., 2004; Duwenig, Steup, Willmitzer, & Kossmann, 1997; Schopper et al., 2015). Mutants that lack DPE2 are compromised in starch turnover in darkness and show a starch excess phenotype (Chia et al., 2004). In potato, reduction of *PHS2* expression results in early flowering and increased sprouting, possibly indicating modification of sugar signaling (Schopper et al., 2015). In sorghum stems, genes encoding DPE2 and PHS2 show 37‐fold and 10‐fold increases in expression in parallel with stem sucrose and starch accumulation, DPE and PHS may be involved in synthesis and remobilization of sucrose from the stem, where they catalyze intermediate steps between maltose metabolism and sucrose synthesis (Lu & Sharkey, 2004).

### 4.2 Regulation of stem starch gene expression and accumulation

The sucrose transported to stems is utilized for many purposes that depend on growth and development. During rapid stem elongation, sucrose is used principally for cell wall biosynthesis and only low levels of sucrose and starch accumulate in elongating internodes under good growing conditions. Floral initiation reprograms the shoot apical meristem from production of nascent internodes to production of the peduncle and panicle. Nascent internodes produced prior to floral transition grow out between floral transition and anthesis. Stem growth and secondary cell wall formation continues until ~7 days before anthesis maintaining a sink for sucrose transported to stems. However, during the transition from rapid stem growth/secondary cell wall formation and rapid grain filling, there is a reduction in these sink activities that is correlated with the accumulation of stem sucrose and starch. An early step in the transition of stems to becoming heterotrophic storage depots involves the downregulation of vacuolar invertase gene expression starting at floral initiation so that sucrose can accumulate in vacuoles (McKinley et al., 2016a). Stem sucrose sometimes begins to accumulate prior to anthesis but usually reaches maximal levels between anthesis and grain maturity. Some internodes accumulate as a percentage of their biomass, ~10%–12% starch, and ~30% sucrose. Preferential accumulation of sucrose is logical because sequestration and remobilization of sucrose are energetically less costly compared to starch. However, as starch is sequestered as a large polymer with low osmotic activity, accumulation of starch can occur in pith cells that already have high levels of vacuolar sucrose providing an additional, longer term store of sugar for grain filling or tillering (ratooning) post‐anthesis.

As the induction of the starch biosynthesis pathway in stems is correlated with the accumulation of sucrose, it is likely that the expression of genes involved in stem starch metabolism is regulated by stem sucrose levels directly or indirectly. Follow on studies will be needed to determine whether sucrose/glucose‐sensing pathways (i.e., T6P, SnRK1, TOR, hexokinase) regulate the expression of genes in the stem starch pathway. Additional levels of regulation that occur in chloroplasts and amyloplasts (i.e., metabolic feedback, redox) (Cho et al., 1999; Mikkelsen et al., 2005; Valerio et al., 2011) may also be important modulators of starch accumulation and turnover in stems.

### 4.3 Summary

In this study the temporal dynamics and spatial distribution of starch accumulation in stems of the sweet sorghum Della was characterized. Transcriptome and phylogenetic analyses helped identify differentially expressed genes involved in stem starch metabolism and sugar transport through the outer membrane of amyloplasts. Specific gene family members involved in stem starch metabolism were differentially expressed in stems and leaves.

## AUTHOR CONTRIBUTIONS

B.A.M. and J.E.M. designed the research; B.A.M. and W.L.R. cultivated plant material; B.A.M. and A.L.C. performed experiments; B.A.M. and J.E.M. performed the analysis of data; B.A.M. and J.E.M. wrote the manuscript.
