# Supplementary material for: Developmental dynamics of stem starch accumulation in Sorghum bicolor
Source: Plant Direct. 2018 Aug 20;2(8):e00074. doi: 10.1002/pld3.74 (PMC6508807; doi:10.1002/pld3.74)
Supplement: Supplementary file 1 [file PLD3-2-e00074-s001.docx]

**Figure S1.** Analysis of the expression of plastidial and cytosolic members of the sorghum AGPase large and small subunit gene families.  The phylogeny of the AGPase gene family is shown to the left of the table. Transcripts with various shades of blue and red represent the large and small subunits of AGPase respectively.  Important loci found in maize are noted to the left of the transcript ID.  The column titled cTP represents the prediction results from ChloroP, an algorithm that predicts the presence of chloroplastidial targeting peptides (cTP) in query sequences; Y indicates that a targeting sequence was identified.  APS = small subunit, APL = large subunit.

**Figure S2.** Phylogenetic analysis of sorghum AGPases using genes with validated function from Huang et al. 2014.  Bt2a and b refer to the BRITTLE2 a and b loci in maize. Sh2 refers to the SHRUNKEN2 locus in maize. LS and SS refer to large and small subunits respectively. Y and N next to transcript IDs refer to the existence of a predicted plastidial peptide targeting sequence.

**Figure S3.** Phylogenetic analysis of the sorghum starch synthases using genes with previously validated function (Hirose and Terao, 2004). Protein sequences were clustered using Clustal Omega using default settings. Red gene IDs indicate sorghum genes. SS = starch synthase, GBSS = granule bound starch synthase.

**Figure S4.** Starch metabolism genes that are differentially regulated between stems and leaves. The analysis was limited to transcripts that exhibited a max expression > 10 TPM at any of the time-points included. The samples shaped purple were obtained from the BTx623 expression atlas.

**Supplemental table 1.**Starch biosynthesis transcript expression in the sorghum stem during development. Expression level is represented by transcripts per million (TPM). Increasing green coloration in the heat map represents increasing expression. The maximum fold change (max FC) represents the maximum fold change between any two time-points during the developmental time course. The column min FDR represents the minimum FDR associated with the maximum fold change.

**Supplemental table 2.** Starch degradation transcript expression in the sorghum stem during development. Expression level is represented by transcripts per million (TPM). Increasing green coloration in the heat map represents increasing expression. The maximum fold change (max FC) represents the maximum fold change between any two time-points during the developmental time course. The column min FDR represents the minimum FDR associated with the maximum fold change.

**Supplemental table 3.**The expression of genes families involved in carbohydrate transport in the sorghum stem during development. Expression level is represented by transcripts per million (TPM). Increasing green coloration in the heat map represents increasing expression. The maximum fold change (max FC) represents the maximum fold change between any two time-points during the developmental time course. The column min FDR represents the minimum FDR associated with the maximum fold change.
