# Supplementary material for: Developmental dynamics of stem starch accumulation in Sorghum bicolor
Source: Plant Direct. 2018 Aug 20;2(8):e00074. doi: 10.1002/pld3.74 (PMC6508807; doi:10.1002/pld3.74)
Supplement: Supplementary file 2 [file PLD3-2-e00074-s002.pdf]

Reviewer #1:

In the work entitled "Developmental Dynamics of Stem Starch Accumulation in Sorghum bicolor", the authors analyzed the accumulation of starch in stems of sweet sorghum and used Illumina´s RNAseq to determine if gene regulation involved in starch synthesis, turn-over and transport had a correspondent association with starch and sugar accumulation in stems and leaves. The work is important in order to better understand the genetic regulation involved in starch accumulation increasing this way, the biomass yield, important for forage use and for production of biofuels and bioproducts.
The manuscript is well organized, the results are explained clearly and both the pictures and the graphs are of very good quality.

Major Points:

1. In the first paragraph of the material and methods, please indicate clearly how many plants (can be in average) were collected per sample and how many biological samples were used.

AU response: Starting at line 507 additional clarification was added by stating that “these three plants were used in subsequent analyses”. Additional clarification was added to the second paragraph of the materials and methods by stating that the samples consisted of 10 plants. The third paragraph of the materials and methods states that “three biological replicates were harvested in the morning of each harvest date” and therefore no additional clarification has been added to this paragraph regrading sample number.

2. Still in Material and methods, how many plants per biological sample?

AU response: Please refer to the response to point 1.

Minor points

Material and Methods

3. Page 19 - lines 566 to 567. Please review the paragraph.

AU response: We have reviewed these lines of this paragraph and do not see anything that seems to be inaccurate or unclear. We would be happy to address this point further if desired.

4. Page 20 - line 627. At this time, the authors are verifying the quality of RNA, not of RNA libraries. The same in line 629.

AU response: In the first instance the term “library has been replaced with “sample”. In the second instance we have deleted “per sample” from the text.

Results

5. First paragraph - The manuscript would benefit from a small list of sorghum genotypes under study at this point since we cannot find any listing at the materials and methods as well.

AU response: The sorghum genotypes used in this study are listed on the x-axis of figure 1. The sorghum genotype Della, used for RNA-seq, was noted in the figure legends and in the materials and methods.

6. Page 6 - line 164. The "(genotypes highlighted in green)" ought to be right after ..."sweet sorghum genotypes".

AU response: We have made this change. Also, we have changed “highlighted in green” to “green bars” and have also specified the color of the grain sorghum bars as “orange bars” which directly follows “grain sorghum genotypes”.

7. Page 6- lines 167 - 178. It is hard to separate the work already reported in 2016 and the analysis done by the authors. The second paragraph induces the reader to associate the starch accumulation on stems with the

previous work but based on Fig S1, I would say that the description correspond to the analysis performed for the manuscript. Please, re-write in such a manner that does not misguide the reader.

AU response: We agree that this data could have been presented in a clearer fashion. The goal of Figure 2 and S-Fig 1 in the original text was to show changes in sorghum stem soluble sugars and starch as a function of plant development in the field (Fig 2) and greenhouse (S-Fig 1). Fig 2B (lower graph) in the original text showed the distribution of starch in stem internodes at grain maturity. First, used the data showing the spatial distribution of starch in different internodes of Della plants at grain maturity to create Figure 3. Second, we used data on sugar and starch accumulation presented in the original Fig 2A (field) and S-Fig 1 (greenhouse) to create a new Figure 2 that shows both sets of data (revised text). In the revised text, Figure 2A provides a general description of the progression of plant developmental stages spanned by the experiment. Figure 2B, shows a plot of changes in % of stem dry weight represented by soluble sugars (sucrose, glucose, and fructose) (black line, left axis) and starch (green line, right axis) during plant development (days relative to anthesis, x-axis). Figure 2C shows a similar plot for Della plants grown in the greenhouse. The data shows that sorghum stems accumulate soluble sugars and starch between floral initiation and anthesis with peak accumulation occurring ~30-53 days post-anthesis. Higher stem sugar and starch accumulated in field grown plants most likely due to higher light conditions (as explained in the text). RNA for RNA-seq analysis was obtained from Della plants grown in the greenhouse.

8. Page 6 - line 182. The authors show an image (Fig 2A) without any description in the text. Please, describe briefly figure 2A.

AU response: Figure 2A is now described in the Figure 2 legend and in the text.

9. Fig. 3. Would benefit from some within figure labeling to distinguish the different stem tissues such as the phloem and the xylem. In fig.3 legend, there is a double "near the".

AU response: Different cell types and tissues within the stem are now labeled in Figure 4 (formerly Fig 3). The duplicated “near the” has been removed.

10. Page 11 - lines 324-335. Please indicate the figure letter to the corresponding genes analyzed between stems and leaves.

AU response: The figure letters indicating which genes were analyzed between stems and leaves were inserted for clarity.

Discussion

11. Page 14 - line 413. Please, add "monocot" species....

AU response: The term “monocot” has been added in place of plant as recommended.
